# Tissue‐Level Transcriptomic Entropy Reveals Organ‐Specific Aging Patterns and Predicts Cancer Progression

**DOI:** 10.1111/acel.70696

**Published:** 2026-09-04

**Authors:** Gabriel Arantes dos Santos, José Pedro Castro, Gabriela D. A. Guardia, Filipe F. dos Santos, Alexander Birbrair, Pedro A. F. Galante

**Affiliations:** ^1^ Hospital Sírio‐Libanês São Paulo SP Brazil; ^2^ i3S, Instituto de Investigação e Inovação em Saúde Universidade do Porto Porto Portugal; ^3^ Aging and Aneuploidy Laboratory, Instituto de Biologia Molecular e Celular Universidade do Porto Porto Portugal; ^4^ Department of Biochemistry, Instituto de Quimica Universidade de Sao Paulo São Paulo SP Brazil; ^5^ University of Wisconsin‐Madison Madison Wisconsin USA

**Keywords:** biogerontology, functional genomics, geriatric oncology

## Abstract

Although aging and cancer share complex molecular mechanisms, distinguishing causative factors from byproducts remains challenging. Here, we investigated the role of tissue transcriptomic entropy—a measure of transcriptional disorder—in aging and cancer processes by analyzing RNA‐sequencing data from over 25,000 samples from human and mouse tissues. We found that entropy changes during aging are highly tissue‐specific, with some tissues showing increased entropy while others exhibit decreased or stable entropy levels. Moreover, transcriptomic entropy strongly correlates with age‐related processes, showing positive associations with proliferation, cellular senescence, somatic mutation burden, and cellular reprogramming, whereas it negatively correlates with stemness. In cancer, we observed that primary tumors generally display higher entropy than normal tissue, with its levels further increasing in metastatic stages. Cancer treatment modulated entropy patterns in multiple contexts, with changes suggesting a role for transcriptional complexity in tumor plasticity and therapy resistance. Elevated entropy levels predicted poor survival outcomes in multiple cancer types, suggesting its potential as a prognostic marker. Furthermore, differential expression analysis revealed that entropy‐associated genes are enriched in developmental processes and depleted in metabolic pathways, indicating a possible link to cellular dedifferentiation. Finally, we found increased entropy in various age‐related disorders beyond cancer, suggesting that transcriptomic entropy may be a common feature in age‐related diseases. Our findings establish transcriptomic entropy as a fundamental parameter in aging and cancer progression, offering new insights into disease mechanisms.

## Graphical abstract

Tissue‐level transcriptomic entropy shows organ‐specific trajectories during aging and is elevated in cancers. Across large human cohorts, higher entropy associates with proliferation, senescence, reprogramming, and poorer cancer prognosis. High‐entropy biological processes display cancer‐like programs even in normal tissues, supporting entropy as an integrated marker of molecular aging, tumor progression, and age‐related disease.

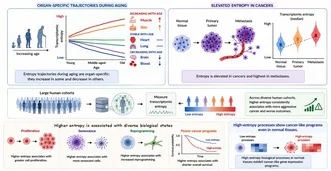

## 1 Introduction

A fundamental challenge in aging research is distinguishing primary causative mechanisms from downstream consequences and compensatory responses (Gems and Partridge 2013; Magalhães and Pedro 2024). While genomic integrity, transcriptional fidelity (Wolters and Schumacher 2013), and epigenetic regulation (Wang et al. 2022) are established determinants of lifespan and healthspan (López‐Otín et al. 2023), systematically quantifying age‐related molecular disorder at the tissue level remains underexplored (Alba‐Linares et al. 2024; Liu et al. 2024).

Information theory, originally developed by Shannon for communication systems (Jones et al. 2024), provides a mathematical framework to measure randomness and uncertainty in biological data. Applied to bulk RNA‐seq, Shannon entropy quantifies the diversity and unpredictability of tissue‐level transcriptional patterns (Conforte et al. 2019). Previous single‐cell studies have demonstrated increased transcriptional entropy in cancer (Teschendorff and Enver 2017), stem cells (Grün et al. 2016; Gulati et al. 2020), and during differentiation (Teschendorff and Feinberg 2021). However, tissue‐level entropy from bulk samples could capture a composite signal reflecting both cell‐intrinsic transcriptional changes and shifts in cellular composition, offering a complementary, population‐scale perspective across large cohorts.

Aging and cancer share a complex, multidirectional relationship (The importance of aging in cancer research 2022; Wolf 2021). While some aging processes oppose carcinogenesis (e.g., stemness decline (dos Santos et al. 2024; Malta et al. 2018) and cellular senescence signatures (Chatsirisupachai et al. 2019)), others advance in parallel (e.g., mutation accumulation (Chatsirisupachai and de Magalhães 2024), inflammation (Leonardi et al. 2018), and loss of tissue identity (Dos Santos et al. 2023)). Transcriptomic and epigenetic clocks have revolutionized aging research (Horvath 2013; Tyshkovskiy et al. 2026), yet the molecular mechanisms driving these changes remain incompletely understood. Recent studies suggest stochastic processes partially explain epigenetic clock dynamics (Tong et al. 2024; Tarkhov et al. 2024; Meyer and Schumacher 2024), and since the epigenome controls transcriptional programs, such changes may propagate to create tissue‐level disorder quantifiable via bulk transcriptomic entropy.

Here, we conduct a large‐scale study of tissue‐level transcriptomic entropy across over 20,000 bulk RNA‐seq samples from cancer and aging contexts. We find systematic increases in transcriptional disorder during tissue‐specific patterns in aging and oncogenesis that associate with age‐related processes including proliferation, senescence, and stemness.

## 2 Results

### 2.1 Aging‐Related Transcriptomic Entropy Variation Is Tissue‐Specific

To understand how entropy changes with aging and cancer, we first calculated Shannon entropy from bulk RNA‐seq data across all GTEx and TCGA samples to quantify tissue‐level transcriptional complexity. Healthy tissues exhibited considerable variation in baseline entropy, with pancreas, blood, kidney, heart, and liver showing the lowest levels, while reproductive tissues (cervix uteri, uterus, testis, ovary) and nerve displayed the highest (Figure S1). Primary tumors showed higher median entropy and greater variability compared to healthy tissues (Figure S1).

Next, we correlated the median entropy of GTEx tissues with tissue turnover using data from Richardson et al. (Richardson et al. 2014). We observed a negative correlation (Figure S1), indicating that tissues with low entropy have slower renewal rates. This finding is consistent with the methylation entropy described by Vaidya et al. (Vaidya et al. 2023), suggesting a potential link between transcriptional disorder and cellular proliferation.

**FIGURE 1:**
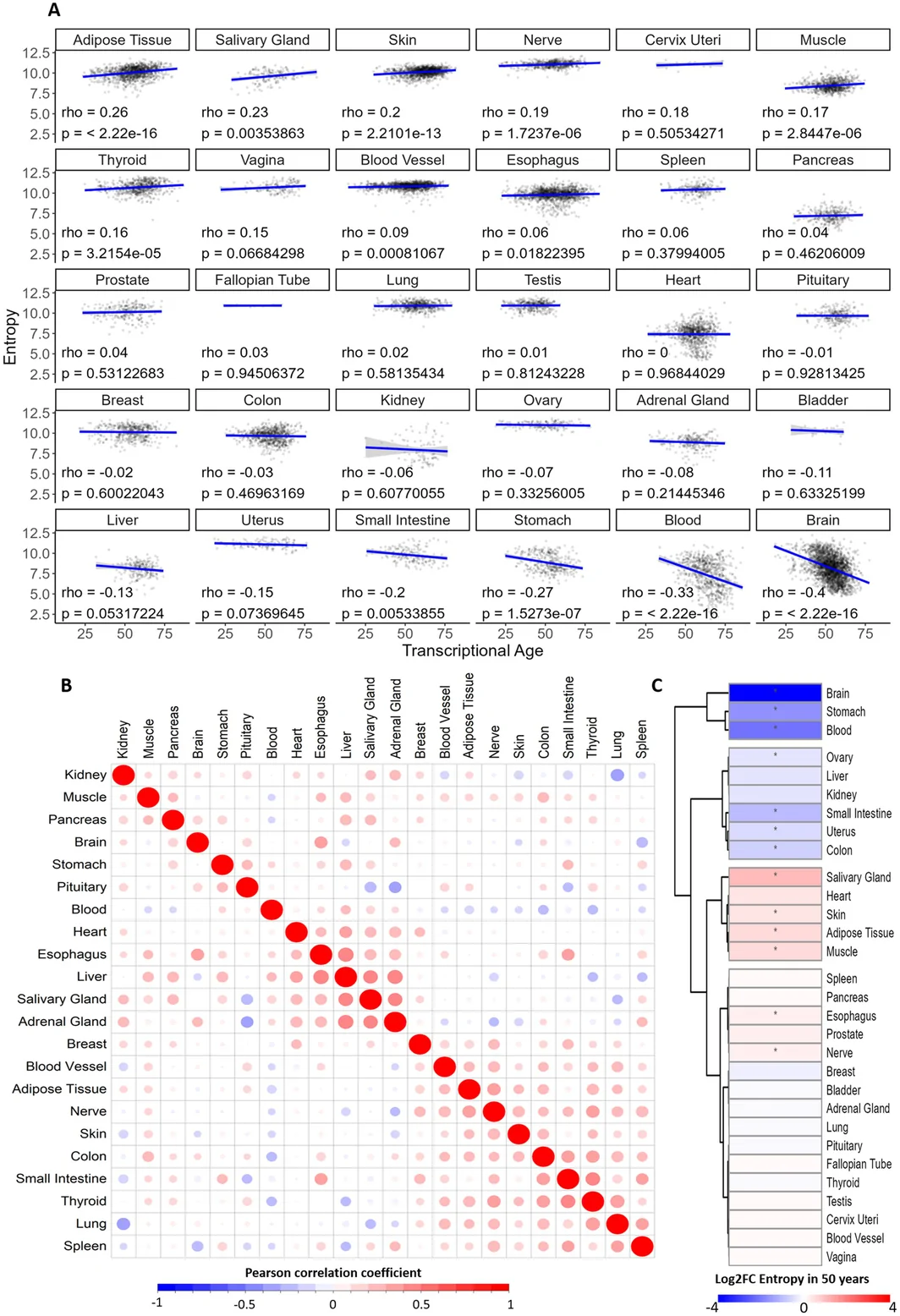
Transcriptomic entropy during human aging. (A) Pearson correlation and linear trend between entropy and transcriptional age in human tissues. (B) Correlogram of entropy values spanning different tissues from the same individual. (C) Heatmap of entropy variation with transcriptional age; results from the adjusted linear model. *p value < 0.05.

To examine age‐related entropy changes, we correlated entropy with transcriptional age across GTEx tissues (Figure 1A). Entropy variation with aging was mostly tissue‐specific: brain, blood, and stomach showed declining entropy, while adipose tissue, salivary gland, and skin exhibited increases. Correlations of entropy across different tissues within the same individual revealed predominantly weak‐to‐moderate associations (Figure 1B), indicating that transcriptional randomness is largely tissue‐autonomous even within individuals.

Since other biological factors can influence gene expression and consequently entropy (Marttila et al. 2020), we used a linear model analysis to adjust entropy variation during aging using available covariates from GTEx. Using this adjusted approach (Figure 1C), we identified three tissue groups: those with declining entropy (brain, stomach, and blood), those with increasing entropy (salivary gland, heart, skin, adipose tissue, and muscle), and those with stable entropy. Chronological age analysis showed similar patterns with attenuated fold changes (Figure S2A). Since GTEx includes some tissues with sub‐tissue regions, we also performed the same analysis for both chronological and transcriptional ages and obtained the same result, which supports organ‐specific variation (Figure S3). These findings demonstrate that bulk transcriptomic entropy does not uniformly increase with aging but instead exhibits tissue‐specific trajectories.

Finally, to assess whether entropy patterns generalize across species, we analyzed brain, kidney, and liver from multiple mammals with varying lifespans (Figure S4). Tissue entropy levels were relatively consistent across species, with no significant correlation between entropy and maximum lifespan, further supporting tissue‐specific regulation independent of aging rate across species.

### 2.2 Transcriptomic Entropy Is Associated With Age‐Related Processes

Since we did not observe a unidirectional relationship between age and transcriptomic entropy across tissues, we investigated whether transcriptomic entropy nevertheless associates with aging‐related processes. We correlated entropy with phenotypes known to be associated with aging: stemness and cell proliferation (both tend to decrease with age) (dos Santos et al. 2024; de Magalhães and Faragher 2008), cellular senescence, and the accumulation of somatic mutations (both tend to increase during aging) (Chatsirisupachai et al. 2019; Chatsirisupachai and de Magalhães 2024).

**FIGURE 2:**
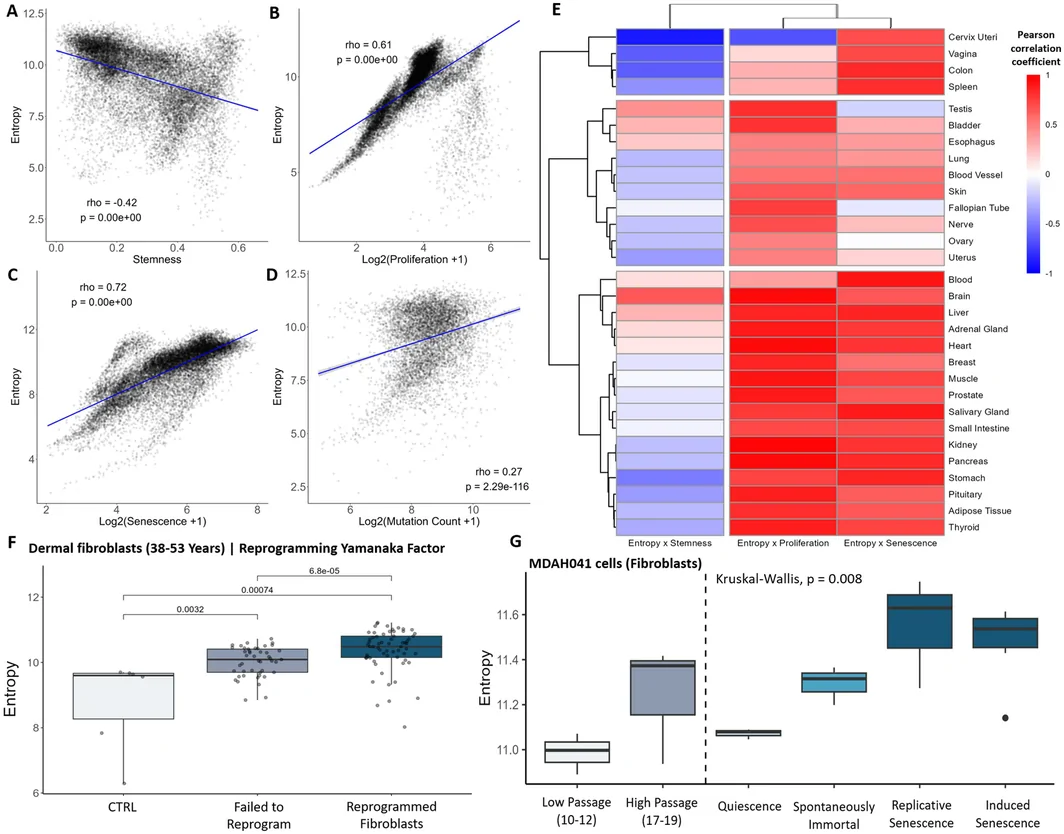
The role of transcriptomic entropy in age‐related processes. (A–D) Pearson correlations between entropy and stemness, proliferation, senescence and mutation count, respectively. Each dot represents a sample. (E) Heatmap of tissue‐specific Pearson correlation coefficients between entropy and stemness, proliferation and senescence. (F) Comparison of entropy in fibroblasts treated with Yamanaka factors (Wilcoxon test): Control, failed reprogramming, and successful reprogramming (data from Gill et al. 2022). (G) Impact of proliferation and senescence on entropy in MDAH041 fibroblasts (data from Purcell et al. 2014). The *p* value represents the global statistical significance.

We observed strong positive correlations between entropy and both proliferation and senescence (rho > 0.6) and a substantial negative correlation with stemness (rho < −0.4) (Figure 2A–D). For approximately 7000 samples with mutation data, we found a trend toward positive correlation with entropy (rho > 0.25). These patterns suggest that transcriptomic entropy associates with biologically meaningful aging‐related processes: reduced regenerative potential (lower stemness), increased cellular cycling, accumulation of senescent cells, and higher mutational burden.

Tissue‐specific analysis revealed distinct patterns across different tissues (Figure 2E). Entropy showed moderate, tissue‐specific associations with stemness, suggesting a more complex relationship in adult tissues. In contrast, proliferation and senescence exhibited strong correlations across most tissues, with notable exceptions in reproductive tissues, which exhibit different cellular dynamics compared to somatic tissues (Brieño‐Enriquez et al. 2022). Similarly, the positive association between somatic mutations and entropy remained consistent across most tissues with available mutation data (Figure S2B).

To explore possible mechanisms underlying transcriptomic entropy, we examined entropy changes during cellular reprogramming induced by Yamanaka factors (Oct4, Sox2, Klf4, c‐Myc), a potential rejuvenation strategy (Figure 2F). Unreprogrammed fibroblasts exhibited lower entropy than Yamanaka factor‐treated cells. Notably, fibroblasts that received treatment but failed to reprogram showed intermediate entropy levels between control and successfully reprogrammed cells. Chemical reprogramming with two (2C) or seven compounds (7C) both increased entropy relative to control mouse fibroblasts (Figure S2C). Interestingly, the less efficient 2C protocol produced higher entropy than the more effective 7C protocol, suggesting that entropy elevation during reprogramming does not scale linearly with reprogramming efficiency. This pattern likely reflects greater transcriptional heterogeneity and instability of partially reprogrammed intermediate cell states in the 2C condition. Finally, analysis of prenatal and postnatal human tissues further supported this finding, revealing higher entropy in the pre‐birth period across most tissues analyzed (Figure S5). In contrast, we observed no consistent entropy differences in response to lifespan‐extending interventions including calorie restriction and rapamycin treatment in mice and humans (Figure S2D–G), suggesting that entropy changes are specific to reprogramming rather than general indicators of longevity extension.

We also examined the paradoxical positive association between entropy and both proliferation and senescence, two mechanistically distinct processes. Analysis of human fibroblasts at different passage numbers revealed that higher passage cells (reflecting increased replication history) exhibited elevated entropy compared to lower passage cells (Figure 2G). Quiescent cells showed lower entropy than immortalized cells, while senescent cells (whether replicatively senescent or chemically induced) displayed the highest entropy in the system. These findings suggest that both cellular proliferation and senescence influence transcriptomic entropy, potentially explaining the tissue‐specific patterns observed in aging.

### 2.3 Increase in Transcriptomic Entropy During Carcinogenesis

**FIGURE 3:**
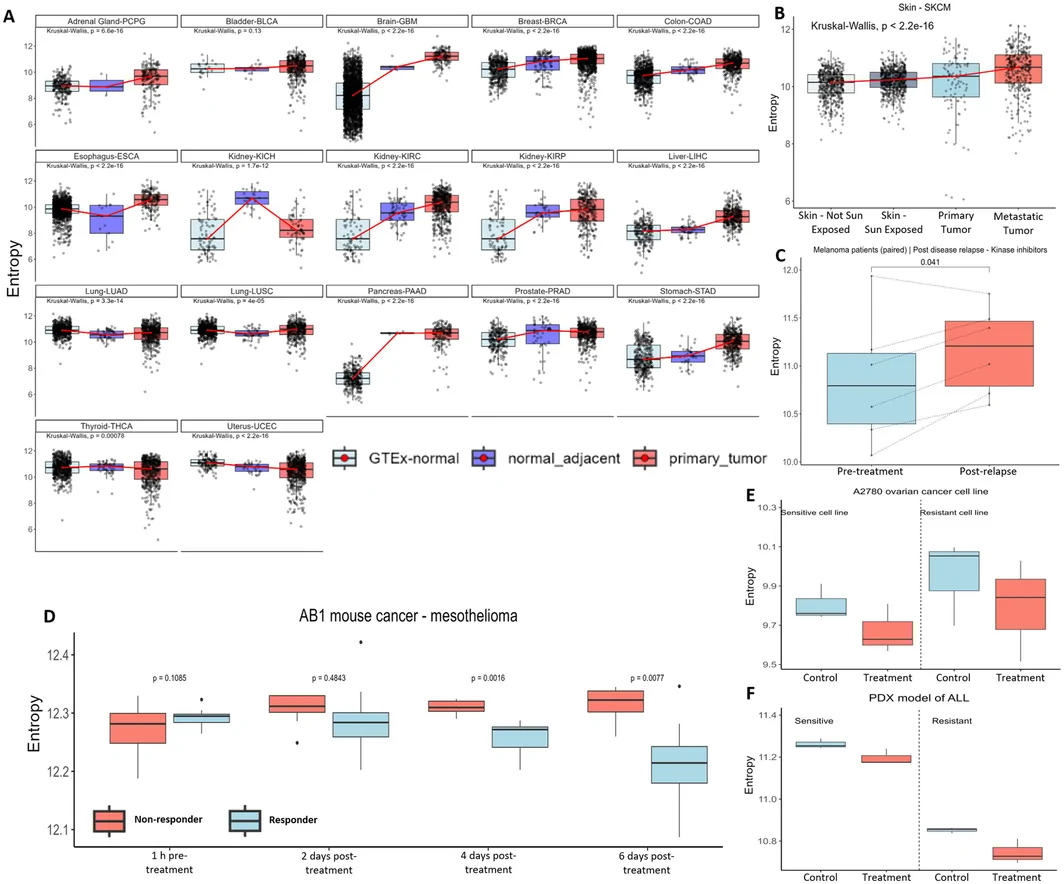
Entropy in carcinogenesis. (A) Comparison of entropy between normal tissue (GTEx), adjacent tumor tissue, and primary tumor tissue (TCGA). (B) Entropy in non‐sun‐exposed and sun‐exposed normal skin (GTEx), and primary and metastatic melanoma (SKCM‐TCGA). Red lines connect the medians for better visualization. (C) Comparison of entropy (Wilcoxon test) pre‐treatment and post‐acquired resistance (paired melanoma samples treated with RAF or RAF + MEK inhibitors), data from Wagle et al. (2014). (D) Comparison of entropy between (Wilcoxon test) responders and non‐responders in mesothelioma (mice) samples treated with immune checkpoint blockade, data from Zemek et al. (2022). (E) Ovarian cancer cell line (sensitive and resistant) data from Golan Berman et al. (2021) treated with cisplatin. (F) Xenograft data from Jing et al. (2018) of ALL (sensitive and resistant samples) treated with dexamethasone and/or decitabine.

Results from the previous section suggest that embryonic‐like states and higher proliferation associate with elevated tissue transcriptomic entropy, which may resemble oncogenic transformation. To examine whether entropy elevation associates with carcinogenesis, we compared normal tissues from GTEx with adjacent normal tissue and primary tumors from TCGA (Figure 3A). In most cases, primary tumors exhibited higher entropy than normal tissues, with notable exceptions in lung tumors (LUAD and LUSC) and lower entropy in thyroid (THCA) and uterine (UCEC) tumors. Adjacent normal tissue occupied an intermediate entropy position, consistent with literature showing that histologically normal tissue adjacent to tumors exhibits intermediate molecular states (Aran et al. 2017). To mitigate concerns about cross‐cohort technical differences, we performed TCGA‐only paired comparisons, confirming that primary tumors exhibit higher entropy than matched adjacent normal tissue across most cancer types (Figure S6), providing support for the association between entropy and carcinogenesis.

To investigate entropy association with cancer progression, we analyzed melanoma, the only TCGA tumor type with an adequate number of metastatic samples. Entropy showed an approximately linear increase from normal not sun‐exposed skin (GTEx) through primary to late‐stage metastatic tumors (Figure 3B). While this comparison involves both GTEx and TCGA samples and should be interpreted with appropriate caution regarding potential technical contributions, the consistent directionality of entropy increase across disease stages suggests an association between transcriptomic entropy and cancer progression.

We next examined treatment responses in paired melanoma samples pre‐ and post‐acquired resistance. 5 out of 6 patients showed increased entropy post‐relapse, with the exception being a patient who already exhibited the highest baseline entropy (Figure 3C). Similar analysis in immunotherapy‐treated mesothelioma revealed that responders exhibited significantly lower entropy than non‐responders (Figure 3D), with a similar but more modest pattern in renal cell carcinoma (Figure S7A). Direct treatment effects in cultured cells further supported these observations: ovarian cancer cell lines treated with cisplatin showed reduced entropy post‐treatment regardless of drug sensitivity (Figure 3E), acute lymphoblastic leukemia xenografts showed entropy reduction following treatment in both sensitive and resistant samples (Figure 3F), and androgen‐pathway inhibitors reduced entropy in prostate cancer models (Figure S7B). These results suggest that treatment acts as a selective pressure, reducing transcriptome entropy through elimination or selection of cell populations.

To validate that entropy elevation in cancers reflects more than compositional changes in cell proportions, we analyzed two independent scRNA‐seq datasets. In liver cancer (LIHC), entropy increased with disease severity, with markedly higher values in primary tumor and metastatic tumors compared to non‐tumor liver (Figure S8A,B). When stratifying by cell type, tumor samples systematically exhibited higher entropy than non‐tumor counterparts within the same annotated cell populations, indicating that entropy elevation is not solely driven by shifts in cell‐type composition but could also reflect cell‐intrinsic transcriptional complexity. In treatment‐naive melanoma patients, metastatic samples showed higher entropy than primary tumors both in pseudobulk analysis and when stratifying by cell type (Figure S8C,D). In patients who developed treatment resistance, entropy increases were specifically detected in malignant cells, macrophages, and CD8+ T cells (Figure S8E), patterns consistent with tumor adaptation and immune remodeling. Together, these single‐cell findings demonstrate that bulk transcriptomic entropy elevation in cancer is not fully explained by cellular composition changes and can be detected within specific cell populations, supporting its use as an informative tissue‐level metric of tumor transcriptional complexity.

Consistent with aging analyses, transcriptomic entropy showed a positive association with somatic mutation burden and tumor mutation burden (TMB) in most tumor types, though with exceptions (Figure S9), suggesting a partial association between entropy and genetic instability in both normal and tumor tissues.

### 2.4 Higher Entropy Is Related to Poor Cancer Survival

To evaluate whether entropy is linked to tumor prognosis, we first examined its association with patient age, as aging alters the molecular landscape of tumors (Chatsirisupachai et al. 2022). In Figure S10, we observe a predominance of negative correlations between patient age and tumor transcriptomic entropy, consistent with reports that tumors in younger patients tend to display more aggressive behavior (Chatsirisupachai et al. 2022), suggesting that higher entropy may be linked to tumor aggressiveness.

**FIGURE 4:**
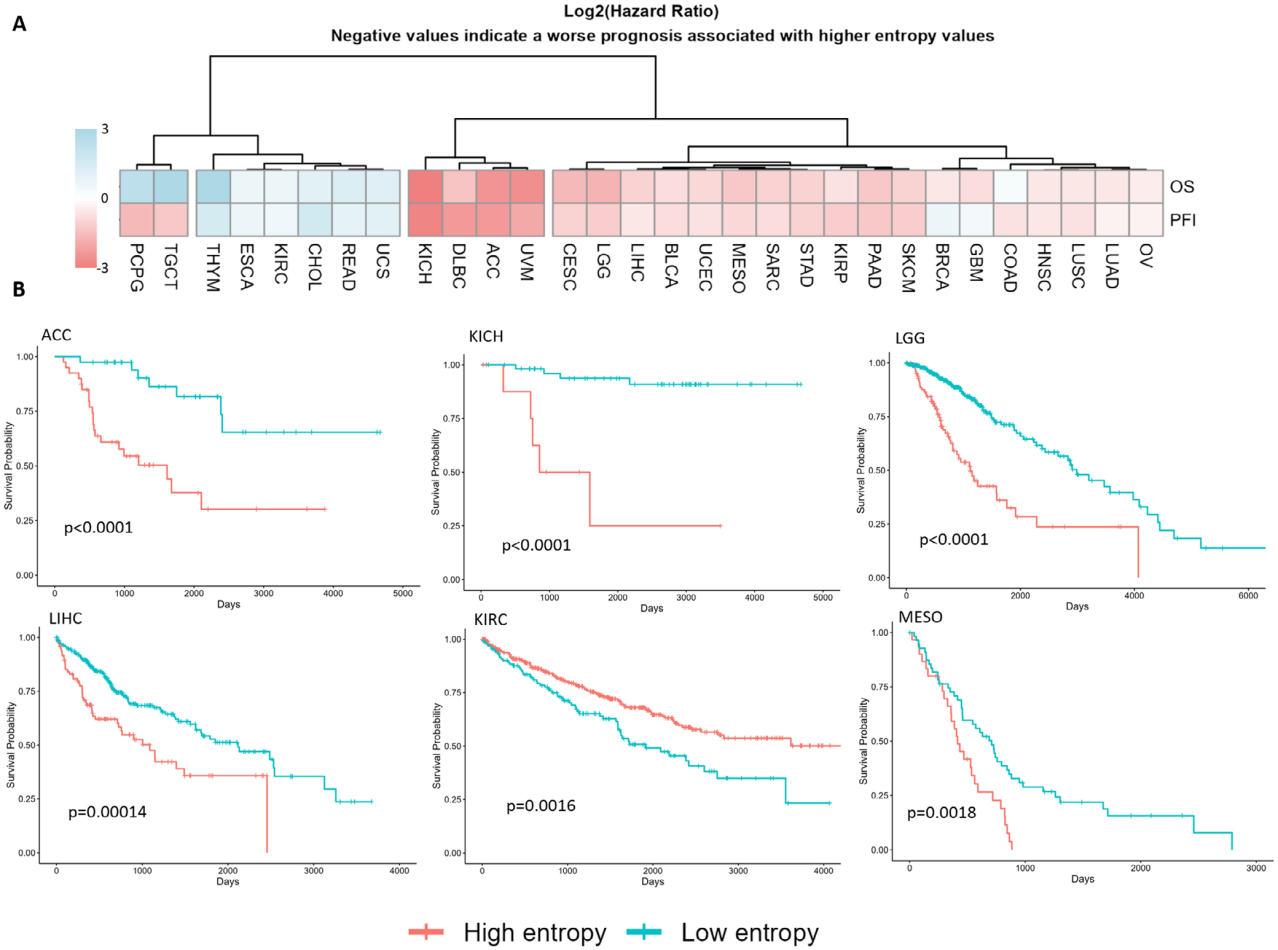
Entropy predicts cancer survival. (A) Heatmap of hazard ratios for overall survival (OS) and progression‐free interval (PFI). Tumors without enough events in OS and/or PFI were excluded. (B) Kaplan–Meier curves of the most significant results (lowest *p* values) for overall survival from the previous heatmap. Data from primary tumors.

We next assessed whether bulk transcriptomic entropy in primary tumors is associated with survival. We stratified patients into high‐ and low‐entropy groups and estimated hazard ratios for overall survival (OS) and progression‐free interval (PFI). Higher entropy was associated with poorer prognosis in most cancer types (Figure 4A). Among the most significant OS associations (Figure 4B), five tumors (ACC, KICH, LGG, LIHC, MESO) showed worse survival in the high‐entropy group, whereas KIRC showed the opposite pattern. Additional significant OS results are shown in Figure S11, where approximately 70% of tumors display poorer survival in the high‐entropy group. For PFI (data not shown), high entropy was similarly associated with worse prognosis in 14 tumor types and with better outcomes in 5 tumor types. Overall, these findings indicate that elevated entropy is frequently, but not uniformly, associated with more adverse prognosis in a cancer‐type‐specific manner.

Since immune‐cell infiltration influences prognosis in many cancers (Jochems and Schlom 2011), we tested whether the survival associations of entropy could be explained primarily by immune composition. Using CIBERSORTx‐based immune deconvolution (Newman et al. 2019) to adjust entropy for inferred immune cell fractions, hazard ratios from composition‐adjusted versus unadjusted entropy remained highly correlated for both OS (rho = 0.92, *p* = 2 × 10^−12^) and PFI (rho = 0.88, *p* = 4.26 × 10^−12^), indicating that the prognostic signal is largely preserved after accounting for immune infiltration.

### 2.5 Enrichment Analysis Links Entropy With Cancer Processes

Our previous results suggest that transcriptomic entropy can be modulated by intrinsic factors (proliferation, senescence, oncogenic transformation) and extrinsic factors (sun exposure, treatment). To investigate whether higher‐entropy samples share a transcriptional program, we performed differential expression analysis relating entropy to protein‐coding gene expression in normal tissues (GTEx) and primary tumors (TCGA).

**FIGURE 5:**
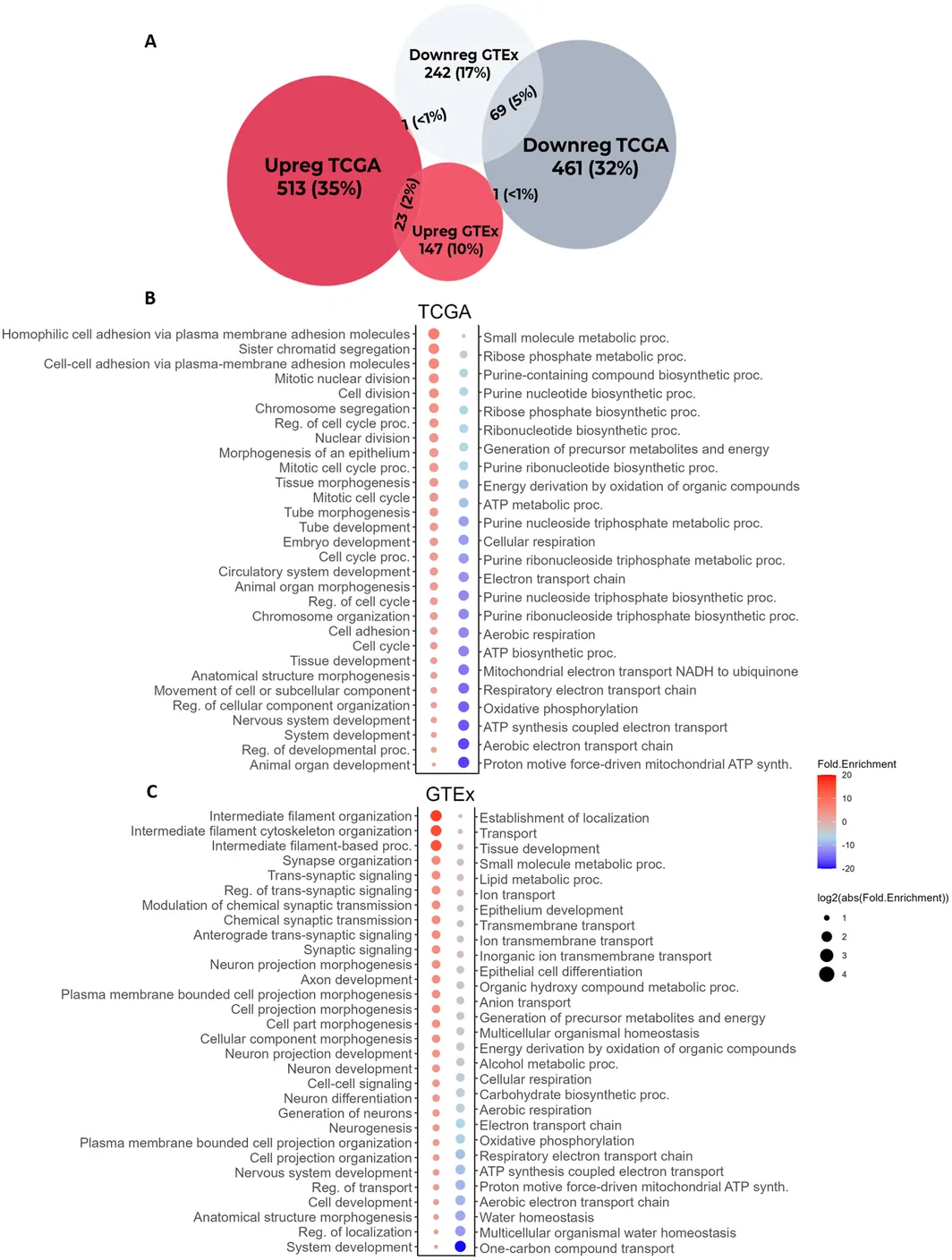
Enrichment analysis for genes with expression variation associated with entropy. (A) Euler diagram of differentially expressed genes in relation to entropy in GTEx and TCGA samples. (B, C) Enrichment of gene ontology biological processes (FDR < 0.05) for upregulated (red) and downregulated (blue) genes from TCGA and GTEX, respectively.

When comparing the overlap of up‐ and down‐regulated genes between GTEx and TCGA, we found that, among 94 genes present in at least two entropy‐associated lists, only two showed opposite directions of change (Figure 5A). This indicates that, despite differences in samples, contexts, and processing, higher‐entropy samples share a conserved expression pattern. To reinforce this notion, we conducted an enrichment analysis for biological processes and observed an interesting result: despite the differences, both normal and tumor tissues show enrichment for developmental and morphogenesis‐related terms, and depletion for genes involved in metabolism and cellular respiration (Figure 5B,C).

This “cancer‐like” enrichment pattern is already present in GTEx normal tissues with higher entropy, consistent with partial dedifferentiation and metabolic reprogramming of adult somatic tissues in a Warburg effect–like manner (Danhier et al. 2017). Thus, even in non‐malignant contexts, higher tissue‐level entropy is accompanied by expression changes that resemble fundamental hallmarks of tumorigenesis.

Given the increase in entropy during partial cellular reprogramming (Figure 2F), we performed a similar enrichment analysis in reprogrammed fibroblasts. Upregulated genes were mainly involved in cell‐cycle processes, resembling the TCGA entropy signature, whereas depleted processes, although partially overlapping with GTEx in transport‐related functions, were otherwise distinct (Figure S12). Overall, these results suggest that reprogramming‐associated entropy shares a proliferation‐related component with cancer but involves gene programs different from those in normal tissues.

### 2.6 Other Age‐Related Diseases Exhibit Similar Increased Transcriptomic Entropy

**FIGURE 6:**
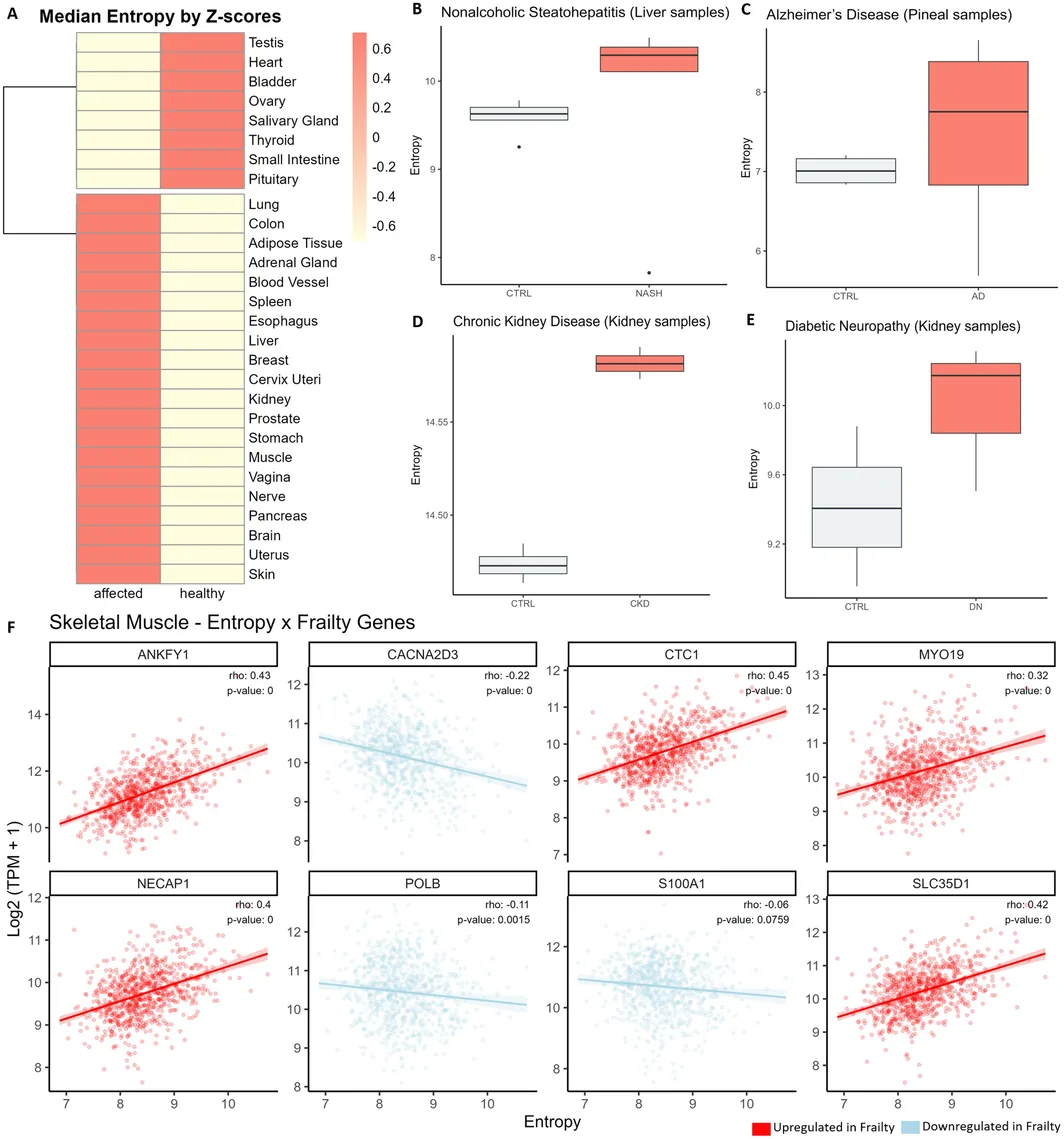
Transcriptomic entropy association with age‐related diseases. (A) *Z*‐score heatmap comparing the median entropy between affected and healthy samples. (B–E) Boxplots showing differences in entropy between control and age‐related diseases: (B) *n* = 5 per group; (C, D) *n* = 4 per group; (E) *n* = 3 per group. CTRL = control. (F) Pearson's correlation between entropy and the expression of frailty genes in GTEx skeletal muscle samples. Red indicates genes that were identified as upregulated in frail patients, and blue indicates those that were downregulated.

Since aging is a major risk factor not only for cancer but also for other chronic diseases (Guo et al. 2022), we asked whether tissue‐level transcriptomic entropy might also be altered in non‐malignant age‐related conditions. Although GTEx classifies its samples as normal, many have pathology reports describing minor alterations (e.g., inflammation, hyperplasia). Comparing median entropy between samples with and without such alterations, we observed that most affected groups showed higher entropy (Figure 6A), suggesting that increased entropy may accompany early subclinical tissue changes.

We then examined mouse models of age‐related diseases, including Alzheimer's disease and diabetic nephropathy. Across four independent datasets, diseased tissues tended to show higher entropy than controls (Figure 6B). Given the small sample sizes, these analyses should be viewed as exploratory, but they are consistent with the possibility that elevated transcriptomic entropy extends beyond cancer to other age‐related pathologies.

Finally, we considered frailty as a marker of morbidity in human aging. Using the eight‐gene frailty panel defined by Baritello et al. (2025) in skeletal muscle, we found that genes upregulated in frail individuals tended to correlate positively with entropy, whereas downregulated genes correlated negatively in GTEx muscle samples. Together with our observation of increased entropy during muscle aging, this pattern further suggests that higher tissue‐level entropy may be linked to age‐related functional decline.

### 2.7 Transcriptional Entropy Is Modulated by Intracellular and Cellular Composition Changes

**FIGURE 7:**
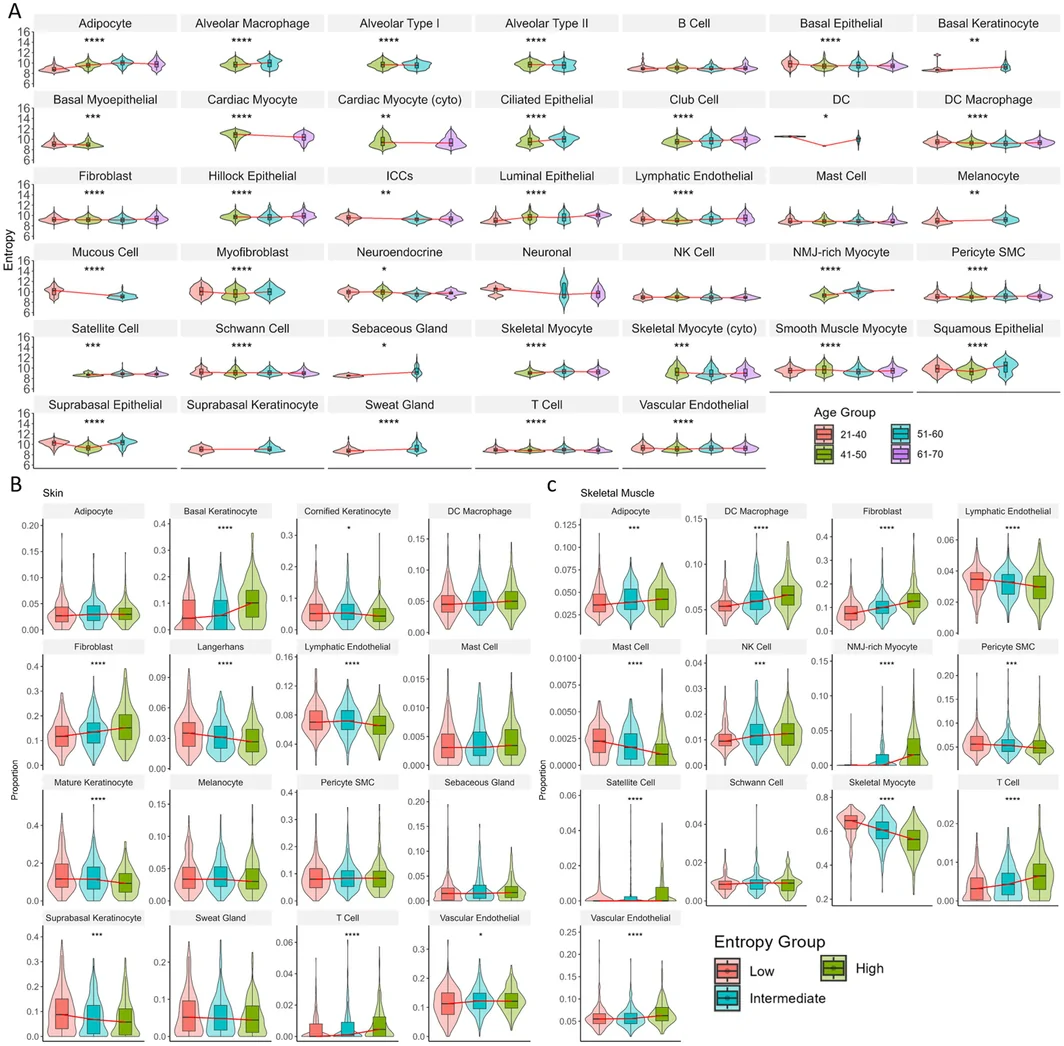
Transcriptomic entropy across several cell types. (A) Entropy levels in each cell type separated by the sample age group (single cell data) from healthy tissues. (B, C) Proportion of cells in each sample, separated by the entropy groups of the skin and skeletal muscle, respectively (deconvolution data). Red lines connect medians to improve visualization. Statistical significance was assessed using the Kruskal–Wallis test for comparisons involving more than two groups and the Wilcoxon rank‐sum test for pairwise comparisons. *p ≤ 0.05; **p ≤ 0.01; ***p ≤ 0.001; ****p ≤ 0.0001.

Bulk RNA‐seq entropy cannot, by itself, distinguish cell‐intrinsic changes from shifts in cellular composition. To test cell‐intrinsic contributions, we analyzed GTEx single‐cell RNA‐seq data from eight tissues and computed entropy per cell. Across cell types, entropy often changed with age in different directions, and each cell type showed a wide entropy range within the same tissue (Figure 7A; Figure S13), indicating that transcriptomic entropy varies both within and between cell populations and is shaped by tissue context and aging.

To investigate compositional effects, we combined bulk GTEx RNA‐seq with deconvolution using Eraslan et al. markers (Eraslan et al. 2022). We focused on skin and skeletal muscle because these tissues show age‐related increases in bulk entropy (Figure 1A). Stratifying samples into low‐, intermediate‐, and high‐entropy groups, we observed marked shifts in inferred cell‐type proportions. In skin, higher entropy was associated with more basal keratinocytes and fewer differentiated keratinocytes; in muscle, with fibroblast expansion and loss of skeletal myocytes, consistent with sarcopenia‐like changes (Larsson et al. 2019). In both tissues, higher‐entropy groups showed overall increased immune and adipocyte fractions (Figure 7B,C), consistent with inflammaging (Lu, Wang, and Benayoun 2022; Agrawal et al. 2023). Similar patterns were seen in other tissues (Figures S14 and S15), indicating that changes in cellular composition substantially influence bulk transcriptomic entropy.

These observations raise the concern that age‐related bulk‐entropy patterns could be largely driven by composition and therefore difficult to interpret biologically. To address this, we regressed entropy on inferred cell‐type proportions and used the residuals as composition‐adjusted entropy. In the eight GTEx tissues with CIBERSORTx‐based deconvolution, cellular composition explained a variable fraction of entropy variance, but age–entropy associations in key tissues such as skin and skeletal muscle remained significant after adjustment (Table S1). Extending the analysis to all GTEx tissues using xCell‐derived scores produced similar results, with most aging–entropy patterns preserved in direction and often in significance (Table S2). Thus, while shifts in cellular composition are an important component of bulk transcriptomic entropy, the age‐related entropy signals we report are not fully reducible to compositional changes, supporting the relevance of tissue‐level entropy as an integrated metric of molecular aging.

## 3 Discussion

The concept of increased disorder impacting aging, longevity and associated diseases is not new and is intuitively accepted due to its logical consistency and supporting evidence (Alba‐Linares et al. 2024; Liu et al. 2024; Ma et al. 2024; Capp and Thomas 2021). For example, several recent studies have shown that methylation changes during aging contain a substantial stochastic component (Tong et al. 2024; Tarkhov et al. 2024; Meyer and Schumacher 2024), and methylation entropy itself has been proposed as a biomarker of aging (Chan et al. 2025). Because epigenetic alterations ultimately act by modulating gene expression (Braunger et al. 2024), it is only natural to ask whether analogous entropy‐based measures at the transcriptomic level are informative. Work on transcriptional drift and noise suggests that expression variability can be both random and structured (Perez‐Gomez et al. 2020), yet at least one study failed to detect a robust increase in transcriptional noise in aged tissues (Ibañez‐Solé et al. 2022), indicating the lack of consensus and the need for further investigation. In this context, we applied Shannon entropy as a bulk metric to thousands of mammalian RNA‐seq samples to characterize how tissue‐level transcriptomic entropy associates with aging and age‐related diseases, particularly cancer.

First, we analyzed how tissue‐level transcriptomic entropy changes with age in human tissues. Most tissues showed stable or reduced entropy, and these trajectories were tissue‐specific, consistent with scRNA‐seq studies in mice (Mao et al. 2023). We further observed that opposing age‐related processes such as senescence and proliferation both associate positively with entropy, which is conceptually similar to the conclusion made by Okada (Okada 2024), and may partially explain the tissue‐specific variation in entropy. Although our analysis provides a broad, cross‐tissue map of aging‐related entropy dynamics, we did not detect clear or consistent non‐monotonic age–entropy patterns within the resolution of our data. The mechanistic basis for this variation with age remains an open question for future, tissue‐focused studies.

We found that tissue‐level transcriptomic entropy increases during cellular reprogramming, a result that may seem counterintuitive given the rejuvenative goal of such therapies, but is consistent with prior reports linking stem‐like states to higher transcriptional disorder (Banerji et al. 2013; Banerji et al. 2015). This pattern further connects the aging and cancer axes, as both reprogramming and tumor progression are associated with elevated entropy (Simpson et al. 2021; Tarabichi et al. 2013). In line with this, we observed that cancer treatments frequently reduce entropy, in agreement with known therapy‐induced transcriptional changes (Xian et al. 2022; Hodge et al. 2023; Cayrefourcq et al. 2021).

Furthermore, our results suggest that transcriptomic entropy may serve as a prognostic biomarker in several tumor types, even after immune infiltration adjustment, and could aid in understanding—and potentially predicting—treatment responses, paving the way for its application in personalized medicine, as previously proposed by Conforte et al. (2019). We also observed an increase in entropy in other age‐related disorders, which could be further explored in future studies, as stochastic molecular processes have already been associated with these types of conditions (Markov et al. 2024; Chen et al. 2022).

Although Shannon entropy quantifies transcriptional randomness, we identified a reproducible gene expression signature associated with higher entropy in both GTEx (normal) and TCGA (primary tumor) samples. These entropy‐associated genes are enriched for pluripotency processes and depleted for metabolic pathways, patterns consistent with cellular dedifferentiation and metabolic reprogramming, hallmarks of carcinogenesis (Hanahan 2022). The entropy‐associated expression signatures may reflect either cellular responses to entropy‐inducing factors (e.g., integrated stress responses (Avelar et al. 2024)) or arise because entropy correlates with other cellular characteristics. Given the associations we observed between entropy and proliferation and senescence, our data are more consistent with the latter explanation, though additional work is needed to fully understand these mechanisms. Our findings further align with the concept of exdifferentiation, in which aging cells progressively lose their differentiated identity (Yang, Hayano, et al. 2023; Lu et al. 2023), and with the role of senescent cells in promoting carcinogenesis through the SASP (Faget et al. 2019; Lee and Schmitt 2019). Together, these observations raise the possibility that tissue‐level entropy may serve as an indicator linking aging‐related transcriptional changes to early carcinogenic processes.

An important consideration is that Shannon entropy, originally a measure of disorder in physical systems, may in our bulk RNA‐seq context capture not only disorder per se but also transcriptomic complexity and cellular heterogeneity. Bulk transcriptomic entropy reflects a composite signal arising from heterogeneity within cell types, diversity across cell types, and shifts in cellular composition. For example, oncogene‐induced senescence is accompanied by increased gene expression heterogeneity (Vasilopoulos and Martínez‐Zamudio 2024), and aged mouse muscle exhibits increased transcriptional diversity alongside functional decline (Liu et al. 2025), patterns consistent with our observations of elevated entropy during senescence and muscle aging. Critically, our deconvolution and single‐cell analyses address the concern that bulk entropy variations could be driven primarily by changes in cellular composition. Using CIBERSORTx and xCell‐based deconvolution, we found that cellular composition explains a variable but often modest fraction of entropy variance, and age‐related entropy increases in key tissues such as skin and skeletal muscle remain significant after adjusting for inferred cell‐type proportions. Furthermore, single‐cell analyses in liver cancer and melanoma show that entropy increases within the same annotated cell types, not solely due to compositional shifts. Together, these results indicate that while cellular composition is an important component of bulk transcriptomic entropy, our measure also captures biologically meaningful signals related to transcriptional changes associated with cancer and age‐related functional decline (Yang, Harrison, and Promislow 2023). Thus, there may exist context‐dependent beneficial and deleterious ranges of entropy: for instance, elevated entropy in pluripotent states and during reprogramming may reflect adaptive plasticity, whereas similar increases in aging tissues may signal loss of identity and functional decline. Distinguishing randomness from biologically meaningful complexity remains a key challenge for future work.

However, there are some limitations to this study. First, our analyses are cross‐sectional and correlational, precluding causal inferences about the role of entropy in aging or disease processes. Second, the analyses conducted here have overlooked the retrotranscriptome, which comprises approximately 50% of our genome and has important implications for both aging and cancer (Gorbunova et al. 2021). Additionally, we do not identify which specific genes contribute most significantly to entropy variation. For instance, a recent study demonstrated that longer genes are more prone to accumulate random damage and alter their expression patterns (Ibañez‐Solé et al. 2023), which is expected to be reflected in transcriptomic entropy and may be particularly relevant for establishing disease‐associated biomarker signatures. Finally, we do not consider diversity at the transcript level (i.e., isoform variation), which may contribute to transcriptomic entropy. This limitation is increasingly relevant given the growing availability of long‐read sequencing technologies, which reveal that we currently underestimate the number of splicing variants of key genes, such as those affecting HER2, potentially contributing to treatment resistance in breast cancer (Guardia et al. 2025). Finally, our use of Shannon entropy, while simpler than more sophisticated entropy metrics developed for single‐cell data (Teschendorff and Enver 2017; Grün et al. 2016; Gulati et al. 2020; Teschendorff and Feinberg 2021; Banerji et al. 2013; Banerji et al. 2015), offers practical advantages for translational application, as it can be readily computed from standard bulk RNA‐seq without requiring specialized single‐cell profiling or complex computational pipelines.

In conclusion, our large‐scale analysis of tissue‐level transcriptomic entropy across thousands of human and mouse samples establishes entropy as a robust, tissue‐specific marker associated with aging and disease progression. We demonstrate that entropy associates with key biological processes including proliferation, senescence, and cellular reprogramming, and frequently predicts cancer prognosis. Our deconvolution and single‐cell validation analyses support the interpretation that bulk transcriptomic entropy captures biologically meaningful signals related to aging‐associated functional decline and tumor progression. These findings suggest that tissue‐level transcriptomic entropy offers new insights into the molecular basis of aging and its relationship to cancer and may serve as a useful metric for understanding age‐related transcriptional changes across diverse tissues and disease contexts.

## 4 Methods

### 4.1 Data Acquisition

Gene expression data from RNA‐seq were obtained from the following sources: GTEx portal (version 8) for healthy human tissues in TPM and read counts (Aguet et al. 2020); GDC portal (https://portal.gdc.cancer.gov/) for TCGA human cancer data (primary tumors, metastatic tumors, and adjacent normal tissue) in TPM and read counts; and GEO database (Clough et al. 2024) for additional human and mouse datasets. Raw data were aligned to GRCh38/hg38 by their respective consortia. Samples lacking complete clinical information were excluded.

For supplementary analyses, we used the following GEO datasets: GSE60340 (senescence and proliferation), GSE165176 (reprogramming), GSE77940 (melanoma pre/post relapse), GSE173201 (ovarian cancer cells), GSE109947 (ALL xenograft), GSE211781 (prostate cancer cells), GSE153941 (mouse tumors), GSE103532 (kidney), GSE230002 (muscle and rapamycin), GSE246954 (chemical reprogramming), GSE120977 (NASH), GSE129586 (Alzheimer's disease), GSE189377 (CKD), and GSE197699 (diabetic nephropathy). For datasets involving genetic editing or treatment, only control samples were analyzed. Additionally, we downloaded prenatal and postnatal human RNA‐seq data from Cardoso‐Moreira et al. (2019) in FPKM.

### 4.2 Shannon Entropy

Shannon entropy is a measure of uncertainty or randomness in a system, widely used to quantify disorder and diversity. In transcriptomics, it assesses the diversity of gene expression by considering how expression levels are distributed among genes. Higher entropy values indicate that expression is more evenly distributed across many genes (greater diversity), whereas lower values indicate concentration in a smaller set of genes (less diversity).

To calculate tissue‐level transcriptomic entropy for each sample, expression levels were first normalized by dividing each value by the total sum of expression for that sample, resulting in relative proportions of gene transcription. Only genes with TPM > 0 were included in the entropy calculation. Using these proportions, we applied the Shannon entropy formula:

$$H=–\sum \left(\mathrm{pᵢ}\times \log_{2}\left(\mathrm{pᵢ}\right)\right)$$

where *pᵢ* represents the proportion of expression for the *i*‐th gene relative to total gene expression in the sample. The summation is over all expressed genes in the sample, yielding a measure of transcriptome diversity.

To assess the technical robustness of this metric, we examined the relationship between entropy and key technical variables. Per‐sample library size (total and aligned reads) showed only negligible correlations with entropy (Table S3), indicating that sequencing depth does not drive entropy estimates. Additionally, we recomputed entropy after restricting calculations to genes expressed in all samples using three different expression thresholds (TPM > 0, TPM > 1, TPM > 10). Entropy values computed on these restricted gene sets remained highly correlated with the original estimates (Table S4), demonstrating robustness to the choice of expressed gene set and indicating that observed entropy variation reflects genuine biological differences rather than technical artifacts.

### 4.3 Chronological and Transcriptional Age

Since GTEx provides age only in broad ranges (20–29, 30–39, 40–49, 50–59, 60–69, and 70–79), we approximated chronological age using the midpoint of each bracket (25, 35, 45, 55, 65, and 75, respectively), as previously described (Dos Santos et al. 2023). To estimate biological age, we calculated transcriptional age for each GTEx sample using the RNAAgeCalc tool (version 1.10.0) (Ren and Kuan 2020) with the “predict_age” function, applying the following parameters: exprtype = “counts”, signature = “all”, and idtype = “ENSEMBL”.

### 4.4 Linear Model

To measure the entropy variation within aging (both chronological and transcriptional), we applied a linear model similar to what was done previously (dos Santos et al. 2024).

For each tissue, entropy fold change (FC) with age was calculated using the model below. To improve visualization, we multiplied the FC value by 50, resulting in the variation of entropy over 50 years. If any variable is not present in the tissue (e.g., sex for vagina or prostate), it is disregarded in the analysis. All information on the subjects was taken directly from the GTEx portal.

$${\mathrm{Yᵢ}}_{j}=\alpha +\beta \left(\mathrm{Age}ᵢ\right)+\gamma \left(\mathrm{Sex}ᵢ\right)+\delta \left(\text{Death}ᵢ\right)+{\mathrm{\varepsilon ᵢ}}_{j}$$

The variables are defined as follows:

- *Yᵢ*_*j*_: The entropy *j* in sample *i*.
- Age*ᵢ*: The age of sample *i* – continuous variable.
- Sex*ᵢ*: The sex of sample *i* – categorical variable.
- Death*ᵢ*: The death classification of sample *i* based on the 4‐point Hardy scale– categorical variable (Ferreira et al. 2018).
- *εᵢ*_*j*_: The error term for entropy *i* in sample *j*.

The variable age was used independently with both chronological and transcriptional age. The linear model was generated using the R package limma (version 3.62.2), using the lmFit() function (Phipson et al. 2016; Ritchie et al. 2015).

### 4.5 Correlogram

To assess entropy correlations among tissues within individuals, we constructed a Pearson correlation matrix using the R package “corrplot (Wei and Simko 2010)” (version 0.95) with hierarchical clustering. For subjects with multiple samples from the same tissue, we used average entropy values. Sexual tissues and those with limited sample sizes (Testis, Prostate, Ovary, Uterus, Vagina, Fallopian Tube, Cervix Uteri, Bladder) were excluded to avoid interfering with hierarchical classification.

### 4.6 Correlation With Mammalian Maximum Lifespan

To correlate transcriptome entropy with maximum lifespan, we used RNA‐seq data from Tyshkovskiy et al. (2023) and Liu et al. (2023), and longevity data from the Human Aging Genomic Resource (de Magalhães et al. 2024). We considered the median entropy for each species/tissue. The maximum lifespan was converted to log10 for better visualization. Outlier species, named in the plot, in each tissue were determined automatically using the ggrepel (version 0.9.6) R package (Slowikowski 2016), with default parameters.

### 4.7 Correlation With Stemness, Proliferation, Cellular Senescence, and Mutation Count

We correlated entropy with aging‐associated biological parameters in GTEx samples. Stemness values were obtained from a previous study (dos Santos et al. 2024). Proliferation was assessed using a validated gene signature derived from the top 1% of genes most positively correlated with PCNA across 36 normal tissues (Ramaker et al. 2017; Venet et al. 2011), calculating the average expression per sample. Senescence was evaluated using the same approach, applying the SenMayo gene list (Saul et al. 2022) by calculating the average expression of all genes within the signature. Somatic mutation counts were obtained from García‐Nieto et al., who estimated mutations for approximately 7000 GTEx samples (García‐Nieto et al. 2019).

### 4.8 Clinical and Molecular Features for Cancer Analysis

To associate tumor entropy with clinical parameters (survival and age), we used the curated data from Liu et al. for the TCGA samples (Liu et al. 2018). TMB and mutation count data were downloaded from the cBioPortal, using the TCGA Pan‐Cancer Study (de Bruijn et al. 2023).

Kaplan–Meier curves and hazard ratio heatmaps were constructed using the R packages survival (version 3.7.0) (Therneau and Grambsch 2013) and survminer (version 0.5.0) (Kassambara et al. 2016). For each cancer type, optimal entropy cutpoints were identified using the surv_cutpoint function (log‐rank test), and Cox proportional hazard models were applied to calculate hazard ratios for overall survival (OS) and progression‐free interval (PFI).

### 4.9 Differential Expression Analysis and Functional Enrichment

To identify differentially expressed genes in relation to entropy, we applied a linear model similar to the one described above for GTEx samples and primary tumors from TCGA. The main differences from before included filtering to evaluate only protein‐coding genes and excluding the “Death” variable for TCGA, while considering chronological age as the “Age” variable in both cases. Furthermore, to achieve pan‐tissue/cancer results rather than tissue‐specific outcomes, we also adjusted the model for tissue (GTEx) or tumor type (TCGA).

Considering that the FC result reflects the variation in expression relative per unit of entropy, we defined differentially expressed genes as those with |Log2 FC| > 0.25 and FDR < 0.05. We used these four gene lists (Upregulated in GTEx or TCGA, Downregulated in GTEx or TCGA) and performed functional enrichment analysis using ShinyGO 0.8 (Ge et al. 2020), with parameters set to species = human, FDR cutoff = 0.05, pathways to show = 30, and pathway database = GO Biological Process. To improve visualization, the Fold Enrichment of downregulated genes was multiplied by −1.

### 4.10 Pathology Slides

To classify GTEx samples as healthy or affected, we downloaded the pathology slide descriptions from the consortium (https://gtexportal.org/home/histologyPage). We manually classified all available samples as healthy if the Pathology Category was empty or labeled as “clean_specimens,” “spermatogenesis” (for testis), “no_abnormalities,” “tma,” or “clean_specimens, no_abnormalities”. The other samples were classified as affected and represent alterations noted by the pathologist, such as fibrosis, congestion, atrophy, hyperplasia, inflammation, etc.

### 4.11 Frailty Genes

To identify genes whose expression reflects frailty, we utilized an 8‐gene panel derived from Baritello et al. (Baritello et al. 2025). Briefly, 63 patients scheduled for cardiac surgery were assessed for mobility, gait speed, and strength and classified as frail. RNA sequencing of muscle biopsies identified ten differentially expressed genes associated with frailty considering the three parameters. For our analysis, we selected the eight protein‐coding genes from this panel: five upregulated (*ANKFY1*, *CTC1*, *MYO19*, *NECAP1*, and *SLC35D1*) and three downregulated (*CACNA2D3*, *POLB*, and *S100A1*) in frail individuals. We extracted the expression of these eight genes using skeletal muscle samples from GTEx to investigate their correlation with transcriptomic entropy (Pearson correlation analysis) to determine if transcriptomic entropy is linked to this frailty‐associated gene signature.

### 4.12 Single Cell RNA Sequencing Analyses

For cancer single‐cell analyses, we used processed and annotated scRNA‐seq datasets from Lu et al. (liver cancer) and Jerby‐Arnon et al. (melanoma) (Lu, Yang, et al. 2022; Jerby‐Arnon et al. 2018). We used the cell type annotations provided by the original studies and computed single‐cell transcriptomic entropy for each cell using Shannon entropy, adding a small constant (1e – 10) to avoid log(0). Entropy values were then summarized by disease stage and by cell type within each condition (Table S8).

For aging analyses, we used GTEx single‐cell RNA‐seq data (version 8) (Aguet et al. 2020), which includes 8 tissues (breast, esophagus mucosa and muscularis, heart, lung, skeletal muscle, prostate, and skin) with samples from 3 subjects per tissue. We applied the same per‐cell entropy calculation as above. Cells annotated as “Unknown” and cell types present in only one age group were excluded from comparisons shown in Figure 7A.

### 4.13 Bulk RNA‐Seq Deconvolution Analyses

For the deconvolution analyses, we obtained bulk RNA‐seq and scRNA‐seq gene count data from GTEx (version 8) (Aguet et al. 2020), and tissue‐specific cell type gene markers from Eraslan et al. (2022). We used the web‐based CIBERSORTx platform (Newman et al. 2019), which enables the *in silico* estimation of cell type proportions in bulk RNA‐seq data by leveraging gene expression signatures derived from single‐cell data. First, for each GTEx tissue with available scRNA‐seq data (breast, esophagus mucosa and muscularis, heart, lung, prostate, skeletal muscle, and skin), we generated a custom cell‐type signature matrix using the “Create Signature Matrix” module with default parameters. Then, using the “Impute Cell Fractions” module, we estimated the relative abundances of each cell type in the corresponding bulk RNA‐seq data, applying the tissue‐specific signature matrices with default parameters, enabling S‐mode batch correction and 100 permutations to assess statistical confidence.

For analyses requiring adjustment of transcriptomic entropy for cell‐type composition, we modeled entropy as a function of inferred cell type proportions and used the model residuals as composition‐adjusted entropy. In GTEx tissues with matched single‐cell data, we regressed per‐sample entropy on all CIBERSORTx‐derived cell type fractions and used the residuals to test associations with transcriptional age. To extend this approach to the full GTEx panel, we repeated the procedure using xCell‐derived scores for seven major cell type categories, fitting separate linear models by tissue. For TCGA, we applied the same strategy using CIBERSORTx with the LM22 immune signature matrix (Newman et al. 2019), regressing tumor entropy on inferred immune cell fractions within each cancer type and using the residuals in downstream survival analyses.

### 4.14 Data Analysis

All data and statistical analyses in this study were conducted using base R (version 4.4.2) functions and the ggpubr R package (version 0.6.1) (Kassambara 2016). The plots were built using the ggplot2 (version 3.5.2) (Wickham 2009) and pheatmap (version 1.0.13) R packages (Kolde 2010), unless otherwise specified.

## Author Contributions

Gabriel Arantes dos Santos, José Pedro Castro and Pedro A.F. Galante conceived the study and designed the analyses. Gabriel Arantes dos Santos, Gabriela D.A. Guardia and Filipe F. dos Santos conducted the analyses. Gabriel Arantes dos Santos, José Pedro Castro, Gabriela D.A. Guardia, Filipe F. dos Santos, Alexander Birbrair and Pedro A.F. Galante critically discussed and interpreted the results, making significant intellectual contributions to the final version of the manuscript. Pedro A.F. Galante supervised the study. All authors contributed to the writing of the manuscript and have all read and approved the final manuscript.

## Funding

Gabriel Arantes dos Santos was supported by a fellowship from Fundação de Amparo à Pesquisa do Estado de São Paulo (FAPESP) [2023/11499‐8]. Gabriela D.A. Guardia was supported by a fellowship from FAPESP [2017/19541‐2] and the Young Scientist program, Hospital Sírio‐Libanês. Filipe F. dos Santos was supported by a fellowship from FAPESP [2020/14158‐9]. Pedro A.F. Galante was supported by grants from Serrapilheira Foundation, FAPESP [2018/15579‐8], and the National Council for Scientific and Technological Development (CNPq). José Pedro Castro was supported by Fundação para a Ciência e Tecnologia with an Assistant Researcher CEEC individual (2022.00872.CEECIND).

## Ethics Statement

The authors have nothing to report.

## Consent

The authors have nothing to report.

## Conflicts of Interest

The authors declare no conflicts of interest.

## Supporting information


**Table S1:** Pearson correlation between transcriptional aging and adjusted entropy (CIBERSORT).
**Table S2:** Pearson correlation between transcriptional aging and adjusted entropy (xCell).
**Table S3:** Pearson correlation between transcriptomic entropy and library size.
**Table S4:** Pearson correlation between original transcriptomic entropy and entropy restricted to common expressed genes.

**Figure S1:** (A, B) Entropy distribution in GTEx and TCGA samples, respectively. (C) Correlation between tissue turnover and median entropy in normal (GTEx) tissues.
**Figure S2:** (A) Heatmap of entropy variation with chronological age. Results from the adjusted linear model. *p value < 0.05. (B) Heatmap of tissue‐specific Pearson correlation coefficients between entropy and proliferation, senescence, mutation count and stemness for GTEx samples with mutation data. (C) Entropy in mouse fibroblasts using 7 (repsox, trans‐2‐phenylcyclopropylamine, DZNep, TTNPB, CHIR99021, forskolin, and valproic acid) or 2 compounds (repsox and trans‐2‐phenylcyclopropylamine) for chemical reprogramming. (D, E) Entropy after calorie restriction (CR) in normal human tissues (muscle and kidney). (F, G) Entropy after Rapamycin treatment in normal mouse tissues (kidney and liver). CTRL = Control.
**Figure S3:** Heatmap of entropy variation with chronological (A) and transcriptional age (B); results from the adjusted linear model. *p value < 0.05. Sub‐tissue regions were included in the analysis.
**Figure S4:** Pearson's Correlation between transcriptomic entropy and maximum lifespan in several mammals for brain, kidney, and liver tissues. Species that deviate from the pattern were named.
**Figure S5:** Transcriptome entropy pre‐ and post‐ birth in human tissues.
**Figure S6:** Transcriptome entropy in normal adjacent tumor and primary cancer TCGA paired samples. Differences were assessed using the Wilcoxon rank‐sum test.
**Figure S7:** (A) Comparison of entropy between responders and non‐responders in renal cell carcinoma (mice) treated with immune checkpoint blockade. (B) Androgen‐dependent prostate cancer cell line treated with different drugs (as indicated in the figure) that inhibit the androgenic pathway. DHT = Dihydrotestosterone. VEH = Vehicle used for control.
**Figure S8:** Single‐cell entropy in cancer. (A) Single‐cell transcriptomic entropy across major lineages in the liver. (B) Median single‐cell entropy per lineage in the liver. (C) Pseudobulk entropy in treatment‐naive melanoma patients, showing higher entropy in metastatic versus primary tumors. (D) Median single‐cell entropy per lineage in treatment‐naive melanoma. (E) Single‐cell entropy by lineage in melanoma patients who develop resistance. B/B.cell = B cells; CAF = cancer‐associated fibroblasts; Endo. = endothelial cells; Mal = malignant cells; NK = natural killer cells; T/T.cell = T cells; TCD4 = CD4+ T cells; TCD8 = CD8+ T cells.
**Figure S9:** (A) Heatmap of Pearson's correlation between entropy and patient TMB (non‐synonymous). (B) Significant results from the previous heatmap. (C) Heatmap of Pearson's correlation between entropy and all somatic mutations. (D) Significant results from the previous heatmap. Data from primary tumors.
**Figure S10:** (A) Heatmap of Pearson's correlation between entropy and patient age. (B) Significant results from the heatmap. Data from primary tumors.
**Figure S11:** Kaplan–Meier curves for overall survival for significant results (*p* < 0.05) excluding those already shown in Figure 4B. Data from primary tumors.
**Figure S12:** Enrichment of gene ontology biological processes for upregulated (red) and downregulated (blue) genes from cellular reprogramming (same data as in Figure 2F). In this analysis, we directly compared dermal fibroblasts versus reprogrammed fibroblasts.
**Figure S13:** Transcriptomic entropy levels for each cell type across the 8 tissues analyzed.
**Figure S14:** Proportion of cells in each sample, separated by the entropy groups, for breast and esophagus (mucosa and muscularis), respectively (deconvolution data).
**Figure S15:** Proportion of cells in each sample, separated by the entropy groups, for heart, lung, and prostate, respectively (deconvolution data).

## Data Availability

No new datasets were generated during the current study. All public datasets analyzed are referenced throughout the manuscript.
