# Supplementary material for: Tissue‐Level Transcriptomic Entropy Reveals Organ‐Specific Aging Patterns and Predicts Cancer Progression
Source: Aging Cell. 2026 Sep 4;25(9):e70696. doi: 10.1111/acel.70696 (PMC13543671; doi:10.1111/acel.70696)
Supplement: Supplementary file 1 [file ACEL-25-e70696-s001.docx]

**Supplementary Figure 1. A-B.** Entropy distribution in GTEx and TCGA samples, respectively. **C.** Correlation between tissue turnover and median entropy in normal (GTEx) tissues.

**Supplementary Figure 2. A** Heatmap of entropy variation with chronological age. Results from the adjusted linear model. * p-value < 0.05. **B.** Heatmap of tissue-specific Pearson correlation coefficients between entropy and proliferation, senescence, mutation count and stemness for GTEx samples with mutation data. **C.** Entropy in mouse fibroblasts using 7 (repsox, trans-2-phenylcyclopropylamine, DZNep, TTNPB, CHIR99021, forskolin, and valproic acid) or 2 compounds (repsox and trans-2-phenylcyclopropylamine) for chemical reprogramming. **D-E.** Entropy after calorie restriction (CR) in normal human tissues (muscle and kidney). **F-G.** Entropy after Rapamycin treatment in normal mouse tissues (kidney and liver). CTRL = Control.

**Supplementary Figure 3.** Heatmap of entropy variation with chronological (A) and transcriptional age (B); results from the adjusted linear model. * p-value < 0.05. Sub-tissue regions were included in the analysis.

**Supplementary Figure 4.** Pearson’s Correlation between transcriptomic entropy and maximum lifespan in several mammals for brain, kidney, and liver tissues. Species that deviate from the pattern were named.

**Supplementary Figure 5.** Transcriptome entropy pre- and post- birth in human tissues.

**Supplementary Figure 6.** Transcriptome entropy in normal adjacent tumor and primary cancer TCGA paired samples. Differences were assessed using the Wilcoxon rank-sum test

**Supplementary Figure 7. A.** Comparison of entropy between responders and non-responders in renal cell carcinoma (mice) treated with immune checkpoint blockade. **B.** Androgen-dependent prostate cancer cell line treated with different drugs (as indicated in the figure) that inhibit the androgenic pathway. DHT = Dihydrotestosterone. VEH = Vehicle used for control.

**Supplementary Figure 8. Single‑cell entropy in cancer. A.**  Single‑cell transcriptomic entropy across major lineages in the liver. **B.** Median single‑cell entropy per lineage in the liver. **C.** Pseudobulk entropy in treatment‑naive melanoma patients, showing higher entropy in metastatic versus primary tumors. **D.** Median single‑cell entropy per lineage in treatment‑naive melanoma. **E.** Single‑cell entropy by lineage in melanoma patients who develop resistance. Endo.= endothelial cells; NK= natural killer cells; TCD8= CD8+ T cells; TCD4= CD4+ T cells; Mal= malignant cells; CAF= cancer-associated fibroblasts; B/B.cell= B cells; T/T.cell= T cells.

**Supplementary Figure 9. A.** Heatmap of Pearson’s correlation between entropy and patient TMB (non-synonymous). **B.** Significant results from the previous heatmap. **C.** Heatmap of Pearson’s correlation between entropy and all somatic mutations. **D.** Significant results from the previous heatmap. Data from primary tumors.

**Supplementary Figure 10. A.** Heatmap of Pearson’s correlation between entropy and patient age. **B.** Significant results from the heatmap. Data from primary tumors.

**Supplementary Figure 11.** Kaplan-Meier curves for overall survival for significant results (p<0.05) excluding those already shown in figure 4B. Data from primary tumors

**Supplementary Figure 12.** Enrichment of gene ontology biological processes for upregulated (red) and downregulated (blue) genes from cellular reprogramming (same data as in Figure 2F). In this analysis, we directly compared dermal fibroblasts versus reprogrammed fibroblasts.

**Supplementary Figure 13.** Transcriptomic entropy levels for each cell type across the 8 tissues analyzed.

**Supplementary Figure 14.** Proportion of cells in each sample, separated by the entropy groups, for breast and esophagus (mucosa and muscularis), respectively (deconvolution data).

**Supplementary Figure 15.** Proportion of cells in each sample, separated by the entropy groups, for heart, lung, and prostate, respectively (deconvolution data).
